# Effect of Xiaoyaosan on Colon Morphology and Intestinal Permeability in Rats With Chronic Unpredictable Mild Stress

**DOI:** 10.3389/fphar.2020.01069

**Published:** 2020-07-16

**Authors:** Fengmin Ding, Jiajia Wu, Chenyue Liu, Qinglai Bian, Wenqi Qiu, Qingyu Ma, Xiaojuan Li, Man Long, Xiaojuan Zou, Jiaxu Chen

**Affiliations:** ^1^ School of Basic Medical Science, Hubei University of Chinese Medicine, Wuhan, China; ^2^ School of Traditional Chinese Medicine, Beijing University of Chinese Medicine, Beijing, China; ^3^ Formula-pattern Research Center, School of Traditional Chinese Medicine, Jinan University, Guangzhou, China

**Keywords:** chronic unpredictable mild stress, intestinal permeability, Xiaoyaosan, tight junction protein, 5-HT

## Abstract

**Purpose:**

In our present study, a rat depression model induced by 6 weeks of chronic unpredictable mild stress (CUMS) was established, and we investigated how Xiaoyaosan affects the intestinal permeability of depressed rats and alterations in tight-junction proteins (TJs) involved in this process.

**Methods:**

The rat depression model was established using CUMS for 6 consecutive weeks. A total of 40 healthy male Sprague-Dawley rats were randomly sorted into four groups: the control group, CUMS group, Xiaoyaosan group, and fluoxetine group. All groups, excluding the control group, were subjected to the 6-week CUMS program to generate the depression model. Body weight, food intake, and behaviors were observed during the modeling period. Histopathological alterations of colon tissue were evaluated by hematoxylin-eosin staining (H&E), and mucus-containing goblet cells were detected by periodic acid-Schiff (PAS) staining. The ultrastructural morphology of colonic mucosa was observed by transmission electron microscopy. Furthermore, immunohistochemistry (IHC) and quantitative reverse transcription polymerase chain reaction (qRT-PCR) were used to determine the expression of TJs. The concentrations of 5-hydroxytryptamine (5-HT) in the hypothalamus and colon were also assessed using enzyme-linked immunosorbent assay (ELISA).

**Results:**

Treatment of depressed rats with Xiaoyaosan alleviated depression-like behaviors as demonstrated by increases in the total distance traveled, the number of entries into the central area in the open field test, the duration spent in the central area, and sucrose preference. Xiaoyaosan treatment also increased body weight gain and food intake in depressed rats. Moreover, Xiaoyaosan treatment effectively improved the colonic pathological and ultrastructural changes, upregulated the expression of ZO-1, occludin, and claudin-1 in the colon, and increased 5-HT levels in the hypothalamus and colonic mucosa.

**Conclusions:**

Xiaoyaosan treatment attenuates depression-like behaviors caused by CUMS and ameliorates CUMS-induced abnormal intestinal permeability, which may be associated with the expression of TJs. These results suggest that Xiaoyaosan exerts an antidepressant effect that may be related to an improvement of intestinal barrier function *via* the brain-gut axis.

## Introduction

Depression is a common psychological or emotional disorder that is usually accompanied by a series of symptoms, including depressed mood, sleep disturbances, loss of interest in most activities (Ravina et al., 2006), low excitement for life, and even suicidal ideation (Lackamp et al., 2016). Furthermore, depression is a chronic and life-threatening mental disorder that affects almost 350 million people worldwide and leads to a substantial economic burden and considerable psychological pressure on families and society (Sobocki et al., 2006; Abe-Higuchi et al., 2016). In the 2010 Global Burden of Disease Study, major depressive disorder (MDD) contributed to a significant disease burden all over the world, and by 2050, MDD is expected to be the second leading cause of death and disease (Young et al., 2016). In addition, depression can increase the risks of cardiac dysfunction, cerebrovascular disease, and other underlying mechanisms of mortality. Therefore, effective treatment interventions are necessary for patients with depression. The newest generation of antidepressants includes serotonin-norepinephrine reuptake inhibitors (SNRIs) and selective serotonin reuptake inhibitors (SSRIs), among others (agomelatine, bupropion, mirtazapine, etc.) (Young et al., 2016). However, the existing antidepressant medications are far from optimal because of their limited effectiveness and adverse side effects (Koenig and Thase, 2009; Cipriani et al., 2011). Common adverse effects include withdrawal effects, sexual dysfunction, and weight gain (Rogers et al., 2013). Other adverse effects, such as feeling emotionally numb, sleep-related movement disorders, tremors, and abnormal bleeding (Cartwright et al., 2016), are also common.

A close connection between the central nervous system (CNS) and gastrointestinal (GI) tract as well as a bidirectional interaction system exists, which is considered the “gut-brain axis” (Rhee, 2009). Recently, the gut microbiota and gut-brain axis have been proven to be increasingly associated with early brain development and the occurrence of psychiatric disorders (Maqsood and Stone, 2016). Serotonin and catecholamines are important neurotransmitters produced by intestinal bacteria and can affect the brain and behavior (Cryan and Dinan, 2012). The intestinal barrier is generally thought to consist of microorganisms, an inner mucous layer, epithelial columnar cells and mixed goblet cells, as well as the inner lamina, which provide an environment for several types of immune system cells (Turner, 2009). Hormones released during the stress response, such as cortisol, can modulate the composition and function of the intestinal mucosal barrier to affect intestinal permeability (Maqsood and Stone, 2016). The tight junctions of epithelial cells (ECs) are the main structures of the intestinal mucosal barrier (Li et al., 2018). Tight junctions consist of two proteins: occludin and claudin, both of which are transmembrane proteins. Occludin and claudin form a physical barrier with adjacent endothelial cells to prevent paracellular diffusion. The role of auxiliary proteins [Zonula occludens (ZO) family] is to anchor the transmembrane proteins onto the cytoskeleton (Wen et al., 2014). Increased gut permeability and altered gut microbiota have been widely accepted as relevant to the etiology, progression, and treatment of many neuropsychiatric disorders (Anderson et al., 2016).

Complementary and alternative medicine (CAM) therapies are a variety of medical practices used for health maintenance, disease prevention, and treatment of illness (Yeung et al., 2014a). At present, CAM therapies are commonly used to treat depression due to the limitations of the currently available treatments. A number of studies have been conducted to test the effectiveness of CAM therapies in treating emotional disorders (Qureshi and Al-Bedah, 2013). Therefore, in addition to pharmacotherapy and psychotherapy, CAM therapies are useful for the treatment of depression. Chinese herbal medicine, a cost-effective and potential CAM therapy with lower toxicity, has been widely used to treat depression in Chinese culture (Yeung et al., 2014a; Wang et al., 2017). The most commonly used and recommended herbal formulas for depression include Xiaoyao San, Chaihu Shugan San, Ganmai Dazao decoction, Guipi decoction, Wendan decoction, Banxia Houpu decoction, Jiawei Xiaoyao San, Chaihu Jia Longgu Muli decoction, and Xiaobuxin decoction (Yeung et al., 2014b). There are some systematic reviews on Chaihu Shugan San (Yeung et al., 2014b), Xiaoyao San (Zhang et al., 2011; Man et al., 2014), and Ganmai Dazao decoction (Yeung et al., 2014a). Oral administration is the main method of taking traditional Chinese medicine (TCM), which is mainly absorbed in the GI tract. Therefore, the mechanisms of absorption in the GI tract of TCM that are used to treat mental disorders require further study.

Our previous study evaluated the antidepressant effect of Xiaoyaosan, which showed a regulatory effect on depressive-like behaviors caused by chronic unpredictable mild stress (CUMS) (Guo et al., 2017; Yan et al., 2018). However, few studies of Xiaoyaosan have focused on the regulation of intestinal permeability in rats with CUMS-induced depression. Therefore, considering the gut-brain axis, this study explored the influence of Xiaoyaosan on the intestinal permeability of depressed rats through detection and analysis of tight-junction proteins (TJs) with the aim of providing new insights for future studies on the antidepressant mechanism of Xiaoyaosan.

## Materials and Methods

### Animals and Grouping

**Figure 1:**
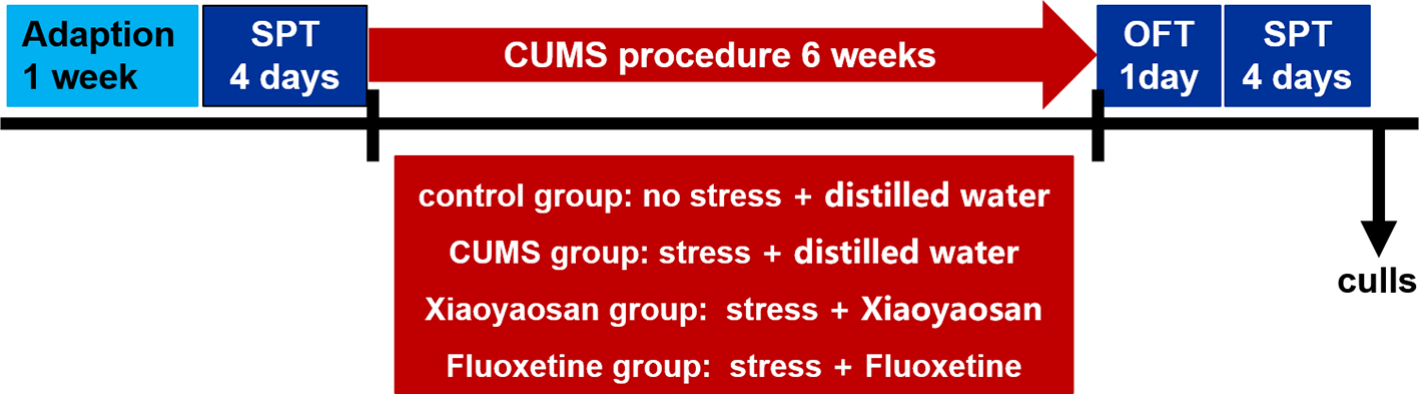
Experimental schedule of this study. After one week of adaption, a baseline sucrose preference test (SPT) was conducted to determine the initial behavioral status of the rats. During the 6-week chronic unpredictable mild stress (CUMS) procedure, the rats in each group received the corresponding treatment associated with their group. An open field test (OFT) and SPT were performed to detect depression-like behavior in rats after CUMS stimulation. The animals were sacrificed, and tissue was collected at the end of behavioral testing.

This study was carried out in accordance with the principles of the Basel Declaration and recommendation of the guidelines for animal welfare (BUCM-4-2017051015-2015) of the Institutional Animal Care and Use Committee of Beijing University of Chinese Medicine. The protocol was approved by the Institutional Animal Care and Use Committee of Beijing University of Chinese Medicine. Forty specific pathogen-free male adult Sprague-Dawley (SD) rats with a body weight of 200 ± 20 g were employed in this study. The animals were provided by Beijing Vital River Laboratory Animal Technology Co., Ltd. (No. SCXK[24]2016-0006 Beijing, China) and were individually housed in an animal room with a barrier system under standard experimental conditions. The rats were randomly sorted into four groups based on initial body weight: the control group, the CUMS group, the Xiaoyaosan group and the fluoxetine group, with 10 rats in each group. The control group had free access to water and food, with no stimulation. The other three groups were subjected to a variety of stressors for six consecutive weeks. The rats were allowed to habituate for 7 d before experimentation. The experimental procedure is shown in **Figure 1**.

### Preparation of Drugs and the Intervention

The Xiaoyaosan Chinese herbal formula comprises eight herbs: Radix Bupleuri (Bupleurum chinense DC. and Bupleurum scorzonerifolium Willd.); Angelica Sinensis (Angelica sinensis (Oliv.) Diels); Rhizoma Atractylodis Macrocephalae (Atractylodes Macrocephala Koidz); Radix Paeoniae Alba (Paeonia lactiflora Pall.); Poria Cocos (Wolfiporia extensa (Peck) Ginns),; Radix Glycyrrhizae (Glycyrrhiza uralensis Fisch.); Rhizoma Zingiberis Recens (Zingiber officinale Rosc.); and Herba Menthae (Mentha haplocalyx Briq.). The Xiaoyaosan powder, provided by Jiuzhitang Co., Ltd. (Changsha, China), was produced based on the procedure described in the Chinese Pharmacopoeia 2015 Edition (National Pharmacopoeia Commission, 2015), and the ratio of different herbs used in production was Radix Bupleuri: Angelica Sinensis: Rhizoma Atractylodis Macrocephalae: Radix Paeoniae Alba: Poria Cocos: Radix Glycyrrhizae: Rhizoma Zingiberis Recens: Herba Menthae=5:5:5:5:5:4:5:1. A brief production process was previously described (Pan et al., 2019). One gram of the powder was obtained from 2.10 g of the raw herbs. Fluoxetine hydrochloride tablets (20 mg/tablet) were obtained from Patheon France (packaged by Lilly Suzhou Pharmaceutical Co., Ltd., Suzhou, China).

According to the 60-kg/d dosage conversion of humans, the rats in the Xiaoyaosan group were given Xiaoyaosan powder dissolved in distilled water at a dose of 2.224 g/kg/d and 0.1 ml/kg bodyweight *via* gavage. The fluoxetine group received fluoxetine dissolved in distilled water at a dose of 3 mg/kg/d and 0.1 ml/kg bodyweight *via* gavage. The rats in the control group and the CUMS group were given the same volume of distilled water. Beginning on the first day of exposure to the stressors, the rats were given distilled water or drugs intragastrically 1 h before stress exposure for 6 weeks.

### UHPLC-LTQ/Orbitrap MS Measure for Xiaoyaosan

The Xiaoyaosan powder was analyzed by a Thermo Scientific Dionex Ultimate 3000 UHPLC Plus coupled to an LTQ/Orbitrap MS system equipped with an operating electrospray ionization source. A 2.1×100-mm BEH 1.7-μm C18 column was equipped for all analyses. The injection volume was 3 μl, the flow rate was 300 μl/min, and the column temperature was 30°C. The mobile phase was a mixture of 0.1% formic acid in water (A) and acetonitrile (B) with negative ion mode: 0–30 min, 95→15% A; 30.1–35 min, 95%–95% A. Other settings were as follows: ion source temperature: 350°C, ionization source voltage: 4 KV, capillary voltage: 35 V, tube lens voltage: 110 V, sheath gas and auxiliary gas: high-purity nitrogen (purity > 99.99%), sheath gas flow rate: 40 arb, and auxiliary air flow rate: 20 arb. Data were acquired using a Fourier transform high-resolution full sweep (TF, Full scan, Resolution 30000); MS/MS uses data-dependent acquisition, and fragmentation was carried out *via* CID. Metabolic profiles were acquired within the range of m/z 100–2000. The Thermo Xcalibur 2.1 workstation (Thermo Scientific, USA) was used to analyze the data.

### CUMS Procedure and Drug Administration

Based on an improved version of a previous study (Shih-Hang et al., 2014), CUMS was induced by daily exposure to alternating stressors for a continuous period of 6 weeks. The stressors included 24 h of food and water deprivation, 5 min of heat stress in an oven at 45°C, 5 min of ice water swimming at 8–10°C, 1 h of noise stimulation at 1,500 Hz and 92 dB, 3 h of body restriction, 2 min of tail pinching, day and night reversal, and other stimuli. To prevent rats from predicting the occurrence of the stimuli, the stressors were randomly distributed, and the same stressors were separated by at least 7 d. The schedule of the induction of CUMS is shown in **Table 1**.

**Table 1:** Schedule of chronic unpredictable mild stress (CUMS) stimulation

Day	Food and water deprivation	Heat stress	Ice water swimming	Noise stimulation	Body restriction	Tail pinching	Foreign object stimulation
Monday	**√**						
Tuesday		**√**					
Wednesday			**√**				
Thursday				**√**			
Friday					**√**		
Saturday						**√**	
Sunday							**√**

### Body Weight and Food Intake

To investigate the effect of the CUMS response on the physical conditions of the rats, the body weight and food intake of each rat were evaluated. Body weight and food intake were recorded every week until the end of the experiment, and values from the same week (1st, 2nd, 3rd, 4th, 5th, and 6th weeks) were compared among the four groups.

### Behavioral Tests

The open field test (OFT) was used to evaluate the locomotor activity and exploratory behavior of the rats. Two hours before the start of testing, the rats were brought to the testing area for adaption, and tests were videotaped and later scored by a trained observer. The test was performed in a 100 cm × 100 cm × 40-cm open square black box. The animals were placed individually in the box and observed for 5 min in a sound-proof environment illuminated with controlled light. The total distance, number of entries into the central area, and duration in the central area during the 5 min were calculated by EthoVision 7.0 software (Noldus)

The susceptibility of the rats to rewards was measured by a sucrose preference test (SPT) to assess the level of depression. The SPT included two periods: training and testing. As previously described (Shih-Hang et al., 2014), after a 72-h training experiment and 24 h of food and water deprivation, the rats were given access to a bottle of pure water and a bottle of 1% sucrose solution simultaneously. One hour later, the volumes of the remaining pure water and sucrose solution were recorded. The SPT was conducted before the induction of CUMS (as a baseline measurement) and after the CUMS experiment was finished.

### Enzyme-Linked Immunosorbent Assay (ELISA) Analysis

Twenty-four hours after the last behavioral test, the rats were deeply anesthetized with chloral hydrate, and after blood collection, the hypothalamus and colon tissue were collected, weighed, and homogenized. The supernatant was collected, and the concentrations of 5-hydroxytryptamine (5-HT) in the hypothalamus and colon were assayed using commercial ELISA kits (Blue Gene For Life Science, Shanghai, China) based on the kit instructions.

### Colon Histology

A sample of colon tissue was excised and fixed in a 4% paraformaldehyde solution. The colon tissue was dehydrated using ethanol and xylene and embedded in paraffin. Then, 5-mm-thick sections were cut. The integrity of the colonic structure was observed using hematoxylin-eosin staining (H&E), and periodic acid-Schiff (PAS) staining was used to detect mucus-containing goblet cells.

### Colon Ultrastructural Morphology

A segment of proximal colon was collected and immediately fixed in 2.5% glutaraldehyde solution at 4°C overnight. Segments were then rinsed three times in a 1 M phosphate-buffered solution for 15 min each time, dehydrated using graded ethanol, soaked in isoamyl acetate twice for 20 min each time, and routinely dried and processed. Colonic ultrastructural images were observed under a Tecnai G2 20, 200-KV electron microscope (FEI, Hillsboro, USA).

### Colon Tissue Immunohistochemical (IHC) Staining

According to a previously described procedure (Yan et al., 2018), paraffin-embedded colonic tissue samples were stained with IHC, and some modifications were made. Colonic sections were first preserved in an oven at 65°C for 2 h to prevent falling and then dewaxed with xylene and hydrated with graded ethanol (100–70%). Then, heat-induced epitope recovery was performed on the slide using the pressure cooker antigen repair method, and the endoperoxidase activity was blocked with 3% H_2_O_2_ for 30 min. Slices were incubated with ZO-1 (1:100), occludin (1:100), or claudin-1 (1:100) antibody overnight at 4°C. Later, the slides used for IHC staining were incubated with horseradish peroxidase (HRP)-conjugated secondary antibodies (anti-rabbit IgG polymer) for 30 min, followed by DAB and hematoxylin staining. Finally, the results of IHC staining were observed and photographed with a microscope (XSP-C204). DAB staining intensity was analyzed using Image Pro Plus 7.0 software (Rockville, USA) and represented by the mean optical density (MOD) value. (MOD=IOD/area, MOD: average response intensity of all selected objects in the visual field. IOD (integrated optical density): sum of the reaction intensities of all selected objects in the entire field of view).

### Western Blot (WB) Analysis

The expression of the ZO-1, occludin, and claudin-1 proteins in the colon was detected by WB. Colon tissues from experimental mice were homogenized in ice-cold RIPA buffer containing protease inhibitors, and the protein-containing supernatants were separated and collected by centrifugation at 10,000 rpm at 4°C for 15 min. Protein concentrations were determined using a bicinchoninic acid (BCA) kit (Thermo Fisher Scientific, Waltham, USA) with bovine serum albumin as a standard. Equivalent amounts of protein from each sample were separated by 10% SDS–PAGE and transferred onto polyvinylidene difluoride membranes. After blocking with 5% defatted milk, the membranes were incubated with primary antibodies overnight at 4°C and then incubated with HRP-conjugated secondary antibodies. The primary antibodies included rabbit anti-ZO-1 (21773-1-AP, Proteintech, 1:500), rabbit anti-occludin (13409-1-AP, Proteintech; 1:1,500), and rabbit anti-claudin-1 (ab15098, Abcam, 1:200). The signals were detected with an enhanced chemiluminescence reagent (Thermo Fisher Scientific, Waltham, USA). The immunoreactive bands were analyzed *via* densitometry using Image Lab (Bio-Rad, California, USA) and standardized to β-actin and were expressed as fold changes relative to the control value.

### Quantitative Reverse Transcription Polymerase Chain Reaction (qRT-PCR)

Total RNA was extracted from colonic tissue using the mirVana™ RNA Isolation Kit (Invitrogen, Carlsbad, USA, AM1561) and then subjected to cDNA synthesis with HiScript II qRT SuperMix (Vazyme Biotech, Nanjing, China, R223-01) in a Geneamp^®^ PCR ABI9700 (Applied Biosystems, Foster City, USA). RT-PCR quantitation was performed using QuantiFast^®^ SYBR^®^ Green PCR Mix (Qiagen, Venlo, Netherlands, 204054) in a LightCycler^®^ 480 II (Roche, Basel, Switzerland). The real-time PCR amplification cycling protocol was as follows: 95°C for 5 min, followed by 40 cycles of 95°C for 10 s and 60°C for 30 s. Each sample was analyzed in triplicate. The primer sequences shown in **Table 2** were designed by Shanghai Generay Biotech Co., Ltd. (Shanghai, China). The target mRNA expression levels were normalized based on the level of the reference gene GAPDH, and then the results were calculated using the 2-ΔΔCt method (Livak and Schmittgen, 2001).

**Table 2:** Primer sequences used in the qRT-PCR analysis.

Gene		Sequence
ZO-1	Forward Reverse	GAGTTTGACAGTGGAGTCG AGCTGAAGGACTCACAGGAA
Occludin	Forward Reverse	TCGGTACAGCAGCAACGATAA CTGTCGTGTAGTCGGTTTCATA
Claudin-1	Forward Reverse	GGTTCATCCTGGCTTCG ATCCACAGTCCCTCGTAG

### Statistical Analysis

SPSS 20.0 software (IBM, Chicago, IL, USA) was used to analyze the data, which were expressed as the means ± standard error of the mean (SEM), and the normality and homogeneity of variance of the data were tested. All statistical analyses were performed using one-way analysis of variance (ANOVA) followed by Fisher’s least significant difference (LSD) test to perform multiple comparisons on the assumption of equal variance. If the data were abnormal or the variance was not uniform, one-way ANOVA with Welch’s robust test of equality of means followed by *post hoc* Dunnett’s T3 test was used to analyze significant differences among groups. ANOVA with repeated measures was used to compare body weights and food intake. A *p*-value < 0.05 was considered statistically significant. Drawing was performed using GraphPad Prism 6.0 software (La Jolla, CA, USA).

## Results

### Quality Control of Xiaoyaosan by UHPLC-LTQ/Orbitrap MS

**Figure 2:**
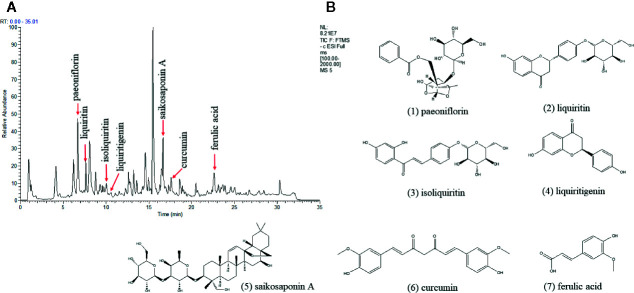
High Performance Liquid Chromatography (HPLC) analysis of ingredients from the Xiaoyaosan sample. **(A)** UHPLC-LTQ/Orbitrap MS chromatogram; **(B)** the molecular structure of paeoniflorin.

The quality control of Xiaoyaosan was investigated by UHPLC-LTQ/Orbitrap MS. The UHPLC-LTQ/Orbitrap MS chromatogram of Xiaoyaosan is shown in **Figure 2A**. Several ingredients in the Xiaoyaosan were identified. As shown in **Figure 2B**, seven compounds were distinguished: (1) paeoniflorin, (2) liquiriti, (3) isoliquiritin, (4) glycyrrhizic acid, (5) saikosaponin a, (6) curcumin, and (7) ferulic acid.

### Xiaoyaosan Treatment Increased Body Weight Gain and Food Intake in the CUMS-Induced Rats

**Figure 3:**
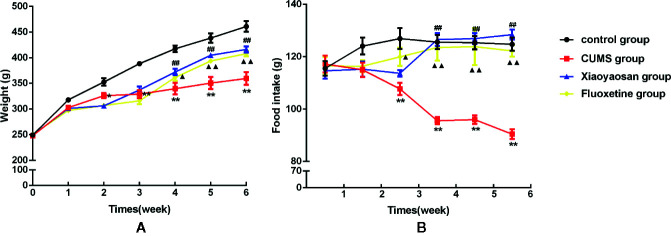
Effects of chronic unpredictable mild stress (CUMS), Xiaoyaosan, and fluoxetine on the body weight gain and food intake of the rats. **(A)** Weekly body weight; **(B)** weekly food intake. The values shown represent the mean ± SEM (n=10). *p < 0.05 or **p < 0.01 versus the control group, ^##^ *p* < 0.01 the Xiaoyaosan group versus the CUMS group, ^▲^ *p* < 0.05 or ^▲▲^ *p* < 0.01 the fluoxetine group versus the CUMS group.

A rat model of CUMS (**Figure 1** and **Table 1**) was established in the present study, and the body weight and food intake of each rat were monitored weekly until the end of the CUMS program to verify whether the CUMS regimen affected these parameters. As shown in **Figure 3A**, over the 6-week experiment, the rats in the control group gained more weight than the rats in any other group. The body weights of the rats in each group did not show a significant difference in the 1^st^ week (*p*>0.05). From the 2^nd^ week until the end of the experiment, body weight appeared to be significantly different between the control and CUMS groups (*p*<0.05 or *p*<0.01). Furthermore, in the 4^th^, 5^th^, and 6^th^ weeks, the weights of the rats in the Xiaoyaosan group were higher than those of the rats in the CUMS group (*p*<0.01), and in the 5^th^ and 6^th^ weeks, the weights of the rats in the fluoxetine group increased significantly compared with those in the CUMS group (*p*<0.01).

Over the 6 weeks of CUMS stimulation, the food intake of the rats in the model group decreased gradually. Furthermore, from the 3^rd^ week until the end of the experiment, weekly food intake was significantly different between the control and CUMS groups (**Figure 3B**, *p*<0.01). In the 4^th^, 5^th^, and 6^th^ weeks, the weekly food intake of the Xiaoyaosan group showed a significant increase compared with that of the CUMS group (**Figure 3B**, *p*<0.01), while in the first three weeks, Xiaoyaosan treatment did not significantly increase weekly food intake compared with CUMS exposure alone (**Figure 3B**, *p*>0.05). From the 3^rd^ week to the 6^th^ week, fluoxetine treatment significantly increased weekly food intake compared with CUMS exposure alone (**Figure 3B**, *p*<0.05 or *p*<0.01).

### Xiaoyaosan Treatment Exerted an Antidepressant-Like Effect on the CUMS-Induced Rats

The OFT and SPT were conducted to measure the eﬀects of Xiaoyaosan on depressive-like behaviors in rats exposed to CUMS.

**Figure 4:**
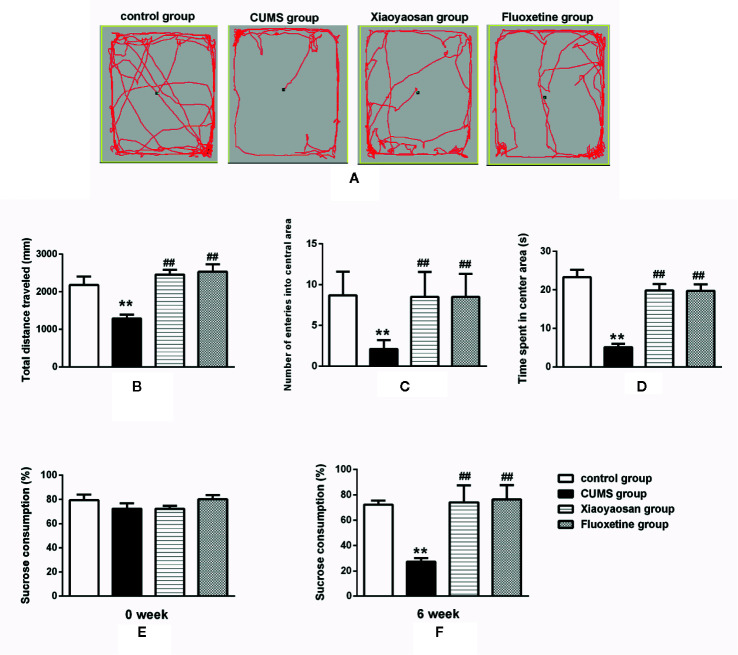
Effects of chronic unpredictable mild stress (CUMS), Xiaoyaosan and fluoxetine on behaviors. **(A)** The motion track in the open field test (OFT). **(B)** The total distance traveled in the OFT. **(C)** The number of entries into the central area of the OFT. **(D)** The time spent in the central area of the OFT. **(E)** Baseline sucrose consumption in the SPT. **(F)** The sucrose consumption rate in the SPT after CUMS stimulation. The values shown represent the mean ± SEM (n=10). **p < 0.01 versus the control group. ^##^ *p* < 0.01 versus the CUMS group.

The OFT results shown in **Figure 4** are as follows: the total distance traveled (B), the number of entries into the central area (C), and the time spent in the central area (D). The aforementioned results in the CUMS group were significantly lower than those in the control group (*p*<0.01). The total movement distance, the number of entries into the central area, and the time spent in the central area were increased after both Xiaoyaosan treatment and fluoxetine treatment (both *p*<0.01, **Figures 4B–D**).

The SPT results are shown in **Figures 4E, F**. Before CUMS exposure, the rats in the four groups exhibited similar sucrose preferences (baseline condition). However, a significant decrease in sucrose preference was noted in the rats exposed to CUMS for 6 weeks (*p*<0.01). After the administration of Xiaoyaosan or fluoxetine, a significant improvement in sucrose preference was observed (both *p*<0.01).

### Xiaoyaosan Treatment Reduced Colonic Inflammation and the Number of Goblet Cells in the CUMS-Induced Rats

**Figure 5:**
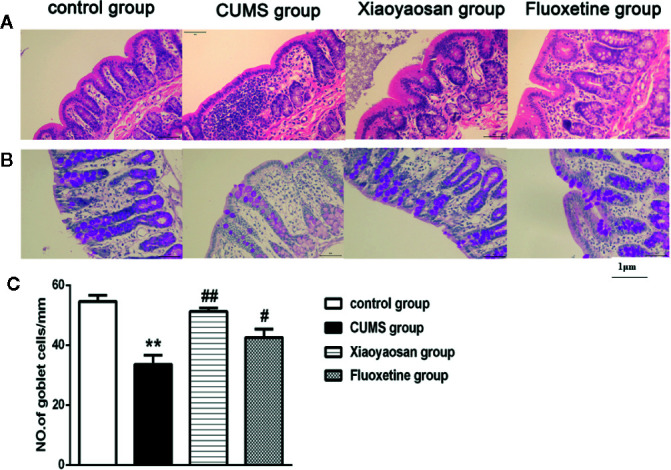
Effects of chronic unpredictable mild stress (CUMS), Xiaoyaosan, and fluoxetine on the colon tissue mucous membrane appearance and goblet cell morphology. **(A)** Sections were stained with hematoxylin-eosin staining (H&E) to assess the mucous membrane appearance. **(B)** Sections were stained with periodic acid-Schiff (PAS) to assess goblet cell morphology. **(C)** Quantification of the mean goblet cells/mm. The values shown represent the mean ± SEM (n=6). **p < 0.01 versus the control group. ^#^ *p* < 0.05 or ^##^ *p* < 0.01 versus the CUMS group. (Magnification: 400×; scale unit: 1 μm).

As shown in **Figure 5A**, the colonic structure of the rats in the control group was intact, and the intestinal glands were well arranged. In the CUMS group, the epithelial structure was destroyed, and the number of intestinal glands decreased or even disappeared. Moreover, infiltration of inflammatory cells was observed in the lamina propria mucosa and muscular layer. Compared with the CUMS group, the infiltration of inflammatory cells and the degree of gland damage were reduced in both the Xiaoyaosan group and fluoxetine group.

As shown in **Figures 5B, C**, after 6 weeks of exposure to CUMS, the number of goblet cells in the colon decreased significantly compared with that in the control group (*p*<0.01), while both Xiaoyaosan treatment and fluoxetine treatments markedly reversed the reduction in goblet cell number (both *p*<0.01).

### Xiaoyaosan Treatment Ameliorated the Colonic Mucosal Ultrastructural Morphology of the CUMS-Induced Rats

**Figure 6:**
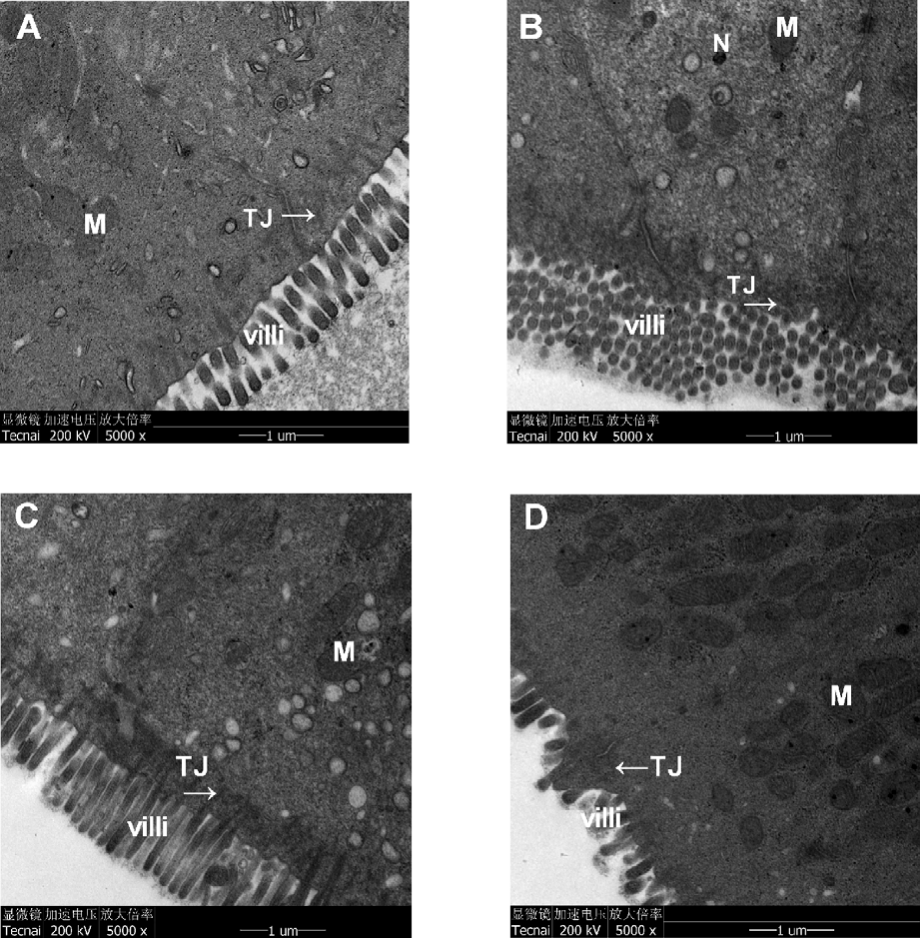
Effects of chronic unpredictable mild stress (CUMS), Xiaoyaosan, and fluoxetine on the ultrastructural morphology of the colonic mucosa of the rats. The epithelium, microvilli and organelle structures in **(A)** the control group, **(B)** the CUMS group, **(C)** the Xiaoyaosan group, and **(D)** the fluoxetine group. M, mitochondria; TJ, tight-junction protein; N, nuclear; villi, microvilli.

Transmission electron micrographs of colonic epithelia demonstrated that the ECs of the control group were regular columnar cells with intact epithelia, orderly microvilli, and tight connections. Furthermore, the ECs exhibited intact organelles with regular nuclei and normal mitochondria, and the surfaces of the ECs were covered with a layer of cell coating (**Figure 6A**). In the CUMS group, the microvilli were reduced in size and number and exhibited an abnormal appearance. Some damage to intercellular junctional complexes (IJCs) occurred, showing a widened intercellular space. Partial disruption or dilatation occurred at the apical parts of the terminal tight junctions. Moreover, little or no cell coating was observed on the surfaces of the ECs (**Figure 6B**). In the Xiaoyaosan group, the size and number of microvilli were increased, and the abnormal arrangement was reversed. Furthermore, the damage to IJCs and the destruction of TJs were notably reduced. In addition, the apoptosis of ECs was also significantly reduced, and more cell coating was present on the surfaces of the ECs than on the ECs in the CUMS group (**Figure 6C**). In the fluoxetine group, the size, number and arrangement of microvilli were also improved; however, the damage to IJCs and the destruction of TJs were not significantly reduced, and less cell coating was observed on the surfaces of the ECs (**Figure 6D**).

### Effect of Xiaoyaosan Treatment on Tight-Junction Proteins in CUMS-Induced Rats

**Figure 7:**
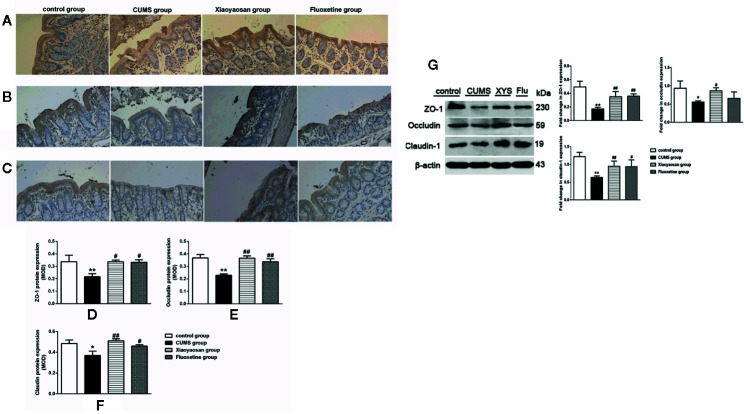
Effects of chronic unpredictable mild stress (CUMS), Xiaoyaosan, and fluoxetine on the expression of tight junction proteins in the colonic mucosa of the rats. Typical micrographs of IHC staining (sections were counterstained with hematoxylin; 400×), and their analyses show the expression of ZO-1 **(A, D)**, occludin **(B, E)**, and claudin-1 **(C, F)** in the colons of the different groups. Immunoblot analysis for the protein levels of ZO-1, occluding, claudin-1 in colon tissue **(G)**. The values represent the mean ± SEM (n=6), and MOD denotes the mean optical density of the areas of interest. *p < 0.05 or **p < 0.01 versus the control group. ^#^ *p* < 0.05 or ^##^ *p* < 0.01 versus the CUMS group.

**Figure 8:**
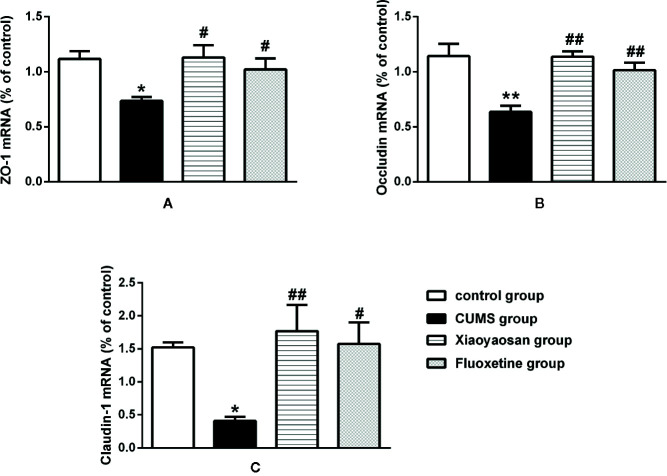
Effects of chronic unpredictable mild stress (CUMS), Xiaoyaosan, and fluoxetine on the mRNA expression of tight junction proteins (ZO-1, occludin, and claudin-1) in the colonic mucosa of the rats. **(A)** The expression of ZO-1 mRNA. **(B)** The expression of occludin mRNA. **(C)** The expression of claudin-1 mRNA. The values represent the mean ± SEM (n=6). *p < 0.05 or **p < 0.01 versus the control group. ^#^ *p* < 0.05 or ^##^ *p* < 0.01 versus the CUMS group.

We performed IHC staining, WB and RT-PCR to assess whether Xiaoyaosan impacted TJ expression levels in colonic ECs. As shown by the IHC and WB results in **Figures 7A–G**, the expression of ZO-1, occludin, and claudin-1 proteins was reduced in the CUMS-treated rats compared to that in the controls (*p*<0.05, *p*<0.01, and *p*<0.05, respectively). Interestingly, the downregulation of ZO-1, occludin, and claudin-1 in the stressed rats was significantly inhibited upon treatment with Xiaoyaosan (*p*<0.05 or *p*<0.01). Fluoxetine treatment also markedly affected ZO-1, occludin, and claudin-1 proteins (*p*<0.05 or *p*<0.01) in the stressed rats. In addition, the RT-PCR results (**Figures 8A–C**) showed that ZO-1, occludin, and claudin-1 mRNA expression levels were reduced in the CUMS group compared to the control group (ZO-1: *p*<0.05; occludin: *p*<0.01; claudin: *p*<0.05). Supplementation with Xiaoyaosan or fluoxetine in stressed rats increased ZO-1, occludin, and claudin-1 mRNA expression in the colon compared with vehicle treatment of stressed rats (*p*<0.05, *p*<0.01, *p*<0.05, respectively). These results suggest that Xiaoyaosan may improve intestinal permeability through the regulation of TJs.

### Xiaoyaosan Treatment Increased 5-HT Levels in the Hypothalamus and Colonic Mucosa of CUMS-Induced Rats

**Figure 9:**
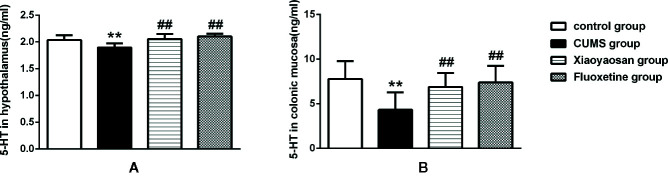
Effects of chronic unpredictable mild stress (CUMS), Xiaoyaosan, and fluoxetine on 5-HT expression in the hypothalamus and colonic mucosa of the rats. **(A)** The concentration of 5-HT in the hypothalamus. **(B)** The concentration of 5-HT in the colonic mucosa. The values shown represent the mean ± SEM (n=10). **p < 0.01 versus the control group. ^##^ *p* < 0.01 versus the CUMS group.

5-HT is closely related to depression and can also regulate GI function. Therefore, we examined the effect of Xiaoyaosan treatment on 5-HT in the hypothalamus and colonic mucosa of CUMS-treated rats. As shown in **Figures 9A, B**, 5-HT levels in both the hypothalamus and the colonic mucosa were decreased in the vehicle-treated stressed rats compared with the controls (both *p*<0.01). Interestingly, supplementation with Xiaoyaosan or fluoxetine in the stressed rats attenuated the decrease in 5-HT levels (both *p*<0.01).

## Discussion

CUMS, a valuable rodent model of depression (Zhang et al., 2010), has been widely used to study depression and evaluate the antidepressant effects of various drugs (Liu et al., 2012). In the CUMS induction procedure, rats were successively exposed to a variety of mild stressors, mimicking the effect of chronic stress on promoting depression and inducing a number of long-term physical, behavioral, neurochemical, and neuroendocrine alterations similar to those observed in depressed patients (Liu et al., 2012). Our previous studies have confirmed that the dose of Xiaoyaosan based on dose conversion of an average adult body weight of 60 kg/d has an antidepressant effect on chronically stressed rats (Jiao et al., 2019). Therefore, according to the daily dosage for adults in clinical practice, the rats in the Xiaoyaosan group were treated with Xiaoyaosan at 2.224 g/kg/d for the intervention in this study. The results of behavioral tests also suggested that Xiaoyaosan at this dose alleviated the depression-like behavior of rats exposed to CUMS. Fluoxetine is a commonly used positive control drug in the study of depression (Zhang et al., 2010; Su et al., 2014), and the dosage of fluoxetine used in our study was based on the clinical adult dosage provided in the drug instructions. The doses of Xiaoyaosan and fluoxetine used in this study were both effective doses. The purpose of the present study was to investigate the effects of Xiaoyaosan on colon morphology and intestinal permeability in rats exposed to CUMS for 6 weeks.

Weight loss is one of the nine specific symptoms of MDD (Sawamura et al., 2016); thus, the animals in this study were weighed weekly to measure how CUMS affected their weights. Some studies have shown an association between depression and food intake (Privitera et al., 2016); therefore, we also recorded the food intake of the rats weekly to observe the effect of CUMS on appetite. We found that after 1 week of CUMS exposure, the stressed rats gained less weight than the unstressed rats. This trend continued until the end of the CUMS procedure, indicating the progression of depression. Both Xiaoyaosan treatment and fluoxetine treatment normalized the slowed weight gain from the 4^th^ week. Furthermore, the food intake of the rats in the CUMS group decreased starting in the 3^rd^ week. In the stressed rats treated with Xiaoyaosan for 3 weeks, an effective improvement in food intake was observed, which may have contributed to their improved weight gain. Similarly, after fluoxetine treatment for 2 weeks, food intake also showed an increasing trend.

The SPT is broadly used to assess the status of anhedonia (Lu et al., 2014). Rats displayed depression-like behaviors with a reduced sucrose consumption preference after exposure to 6 weeks of CUMS, and both Xiaoyaosan treatment and fluoxetine treatment significantly reversed the CUMS-induced anhedonia. The OFT is commonly applied to evaluate the exploratory behavior and general activity of experimental animals in new environments and includes the quality and quantity of activity (Gould, 2010). The total movement distance, the number of times the rats entered the central area and the duration of time spent in the central area were recorded to assess the different behaviors of the rats in each group. The total movement distance is usually used to evaluate locomotor activity to reflect non-anxiety-related behaviors (Choleris et al., 2001). Prolonged exposure to CUMS may affect physiological characteristics of animals, such as locomotion and sleep, and a significant reduction in the total movement distance in the CUMS group was observed in this study. The central area is a new environment for animals in the OFT, and the number of times that a rat enters the central area and the duration of time spent in the central area reflect the exploratory motivation of the rats; loss of interest in new things and a decreased desire to explore are characteristics of depression. We found that the number of times that the rats entered the central area and the duration spent in the central area were notably decreased in depressed rats. Consistent with the results of previous studies (Ding et al., 2017; Yan et al., 2018), our study also confirmed that both Xiaoyaosan treatment and fluoxetine treatment exerted an effective antidepressant effect based on the behavioral test results.

In addition to stress impacting the function of the intestinal barrier, the duration and severity of intestinal barrier dysfunction are dictated by stress exposure during the developmental period (Smith and Clark, 2010; Lennon et al., 2013). Stress has been shown to result in an increase in gut permeability (Söderholm et al., 2002), and studies in humans have also confirmed that acute-stress paradigms can affect intestinal permeability (Tim et al., 2014). Our study demonstrated that stressed rats exhibited impaired intestinal permeability after 6 weeks of CUMS exposure. The observation of impaired intestinal permeability was supported by the following findings: (i) a reduction in mucinogen granules in goblet cells was observed by PAS staining; (ii) profound epithelial structural disruption in the colonic mucosa was observed under transmission electron microscopy (TEM) in stressed rats as evidenced by a widened intercellular space, apoptotic cells and abnormal microvilli; and (iii) marked downregulation of the expression of TJs, including occludin, ZO-1 and claudin-1, was evident in the colonic mucosa. Moreover, after administration of Xiaoyaosan or fluoxetine, the colonic mucosal ultrastructural morphology and TJ expression improved.

Within the past few decades, the importance of the gut–brain axis in regulating stress-related responses has been appreciated (Foster et al., 2017), and more recently, awareness of the crucial role of microbiota in regulating stress-related changes in physiology, behavior, and brain function is increasing (Dinan and Cryan, 2013). A bidirectional communication system between brain and gut microbiota is commonly called the microbiota-brain-gut axis. Increasing evidence suggests that the microbiota-gut-brain axis plays a key role in regulating brain functions, particularly emotional processing and behavior, including those relevant to anxiety and depression (Burokas et al., 2017). In our current study, the results suggested that Xiaoyaosan alleviated the depression-liked behavior of rats induced by 6 weeks CUMS. Whether the antidepressant effect of Xiaoyaosan is correlated with regulation of the gut microbiota composition is unknown. What bacterial products or metabolites from gut commensals influence biological functions? Short-chain fatty acids (SCFAs), the main metabolites produced by bacterial fermentation of dietary fiber in the GI tract, are speculated to have a mediating role in microbiota–gut–brain crosstalk (Dalile et al., 2019). Moreover, SCFAs have a vital impact on intestinal barrier function and inflammation (Bach Knudsen et al., 2018). Improvements in colonic permeability and inflammation by Xiaoyaosan treatment in rats with depression may be related to the production of SCFAs. A recent study demonstrated that Xiaoyaosan regulated the abundance of Bacteroidetes, Proteobacteria, Firmicutes, Chloroflexi, and Planctomycetes in rats with chronic immobilization stress (Zhu et al., 2019). In summary, we propose a hypothesis: the effective mechanism of Xiaoyaosan for both neurological and GI symptoms may involve the production of metabolites (such as SCFAs) through regulation of the composition of the gut microbiota; then, metabolites may translocate from the intestinal mucosa to the systemic circulation where they can interfere with immune regulation and CNS function. More in-depth research is required to explore the mechanism based on metabonomic and microbiological analyses.

The involvement of 5-HT is essential in many physiological and pathological processes of both the CNS and periphery (Mclean et al., 2007). Several studies have provided evidence that impaired 5-HT function in the CNS is indeed linked to depression and susceptibility to depression (Bhagwagar et al., 2002; Cowen, 2008; Liu et al., 2015). The hypothalamic-pituitary-adrenal (HPA) axis is thought to play a mediating role between stress and the development of psychotic symptoms (Thompson et al., 2007), and studies indicate that hyperactivity of the HPA axis is one of the pathogenic mechanisms of depression (Esmaeili et al., 2018; Hooper et al., 2018). Moreover, the hypothalamus is the high-level regulatory center of the axis. Therefore, studying changes in monoamine neurotransmitters in the hypothalamus is important for the antidepressant effect of drugs. In our study, the hypothalamic 5-HT level was decreased in the CUMS-induced rats. Moreover, 5-HT plays a major role in the regulation of GI function (Mclean et al., 2007), and 60–90% of the total 5-HT amount in mammals exists in the GI tract (Thompson, 1971). Therefore, the level of 5-HT in the colonic mucosa was also analyzed in this study. Similarly, the results showed a significant decrease in colonic 5-HT levels in the stressed rats.

Several limitations exist in this study. First, the impact of stress on intestinal permeability is complex and may involve the gut and brain (Thompson, 1971). Studies have indicated that exposure to stress during early life can stimulate plasmatic corticosterone secretion in rat pups and is related to increased intestinal permeability, and this effect appeared to be dominant in the colon (Moussaoui et al., 2014). Moreover, probiotics have been demonstrated to restore colonic tight junction integrity in stressed mice (Ait-Belgnaoui et al., 2014). Although amelioration of abnormal intestinal permeability was observed in the colonic mucosa of the depressed rats after Xiaoyaosan treatment, the potential underlying mechanism requires further investigation. Second, 5-HT has a variety of effects on the enteric nervous system (ENS), including the modulation of smooth muscle function (contraction and relaxation), the promotion of intestinal secretion activities, and responses to visceral pain (Mclean et al., 2007). Nevertheless, the relationship between intestinal permeability and decreased expression of 5-HT in the colonic mucosa of stressed rats requires further elucidation. Finally, although we found that Xiaoyaosan exerts regulatory effects on intestinal permeability and 5-HT levels in the colonic mucosa *in vivo*, the effects of Xiaoyaosan on 5-HT and intestinal barrier integrity *in vitro* remain unknown, and the active ingredients in Xiaoyaosan that affect intestinal permeability or the gut microbiota require further study in the future.

To our knowledge, this study is the first to elucidate the regulatory effect of Xiaoyaosan on intestinal permeability by affecting the expression of TJs in a rat model of CUMS-induced depression. Furthermore, we also provided clear evidence of the antidepressant effects of Xiaoyaosan as shown by an improvement in depression-related behaviors and increased 5-HT levels in the hypothalamus. Notably, Xiaoyaosan treatment also increased 5-HT levels in the colonic mucosa in a rat model of CUMS-induced depression. However, whether the effect of Xiaoyaosan on intestinal permeability is related to 5-HT requires further study.

## Data Availability Statement

This article contains previously unpublished data. The name of the repository and accession number are not available.

## Ethics Statement

All animal experiments followed the guidelines formulated by the Institutional Animal Care and Use Committee of Beijing University of Chinese Medicine and conformed to the guidelines for animal welfare (BUCM-4-2013101501-4001).

## Author Contributions

FD, JW, and JC conceived and designed the experiments. FD, QB, ML, and WQ performed the animal experiment. FD, JW, QM, and XL analyzed the data. CL conducted the UHPLC-LTQ/Orbitrap MS measure for Xiaoyaosan. JW wrote the original manuscript. XZ and JC are primarily responsible for the final content.

## Funding

This research work and publication were supported by National Natural Science Foundation of China under grant numbers 81630104, 81473597 and 81973748, and Huang Zhendong Research Fund for Traditional Chinese Medicine of Jinan University.

## Conflict of Interest

The authors declare that the research was conducted in the absence of any commercial or financial relationships that could be construed as a potential conflict of interest.
