# Supplementary figures and images for: Effect of Xiaoyaosan on Colon Morphology and Intestinal Permeability in Rats With Chronic Unpredictable Mild Stress
Source: Front Pharmacol. 2020 Jul 16;11:1069. doi: 10.3389/fphar.2020.01069 (PMC7378849; doi:10.3389/fphar.2020.01069)

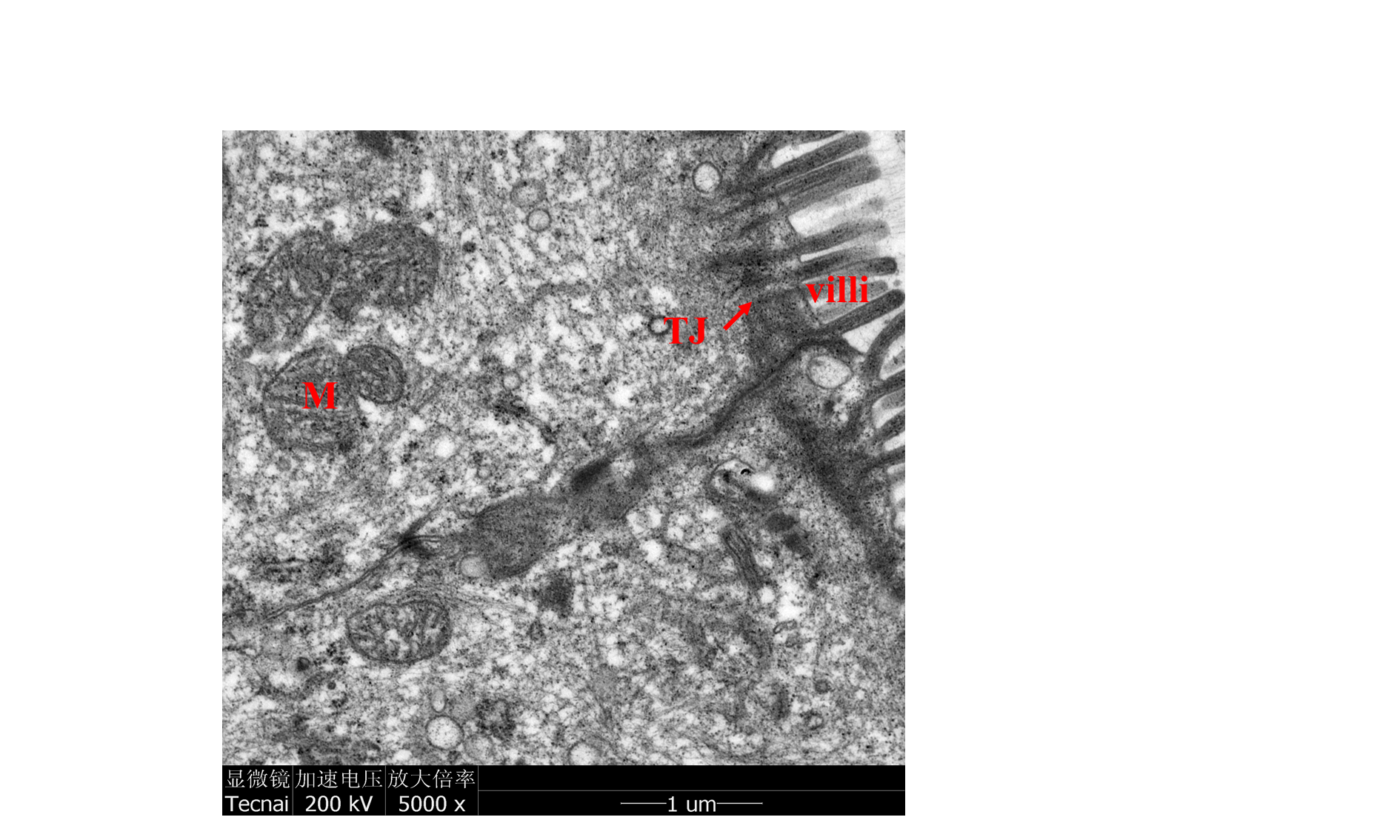

Supplement: Supplementary file 1 [file Image_1.tif]
